# Supplementary material for: Linking functional traits and demography to model species-rich communities
Source: Nat Commun. 2021 May 11;12:2724. doi: 10.1038/s41467-021-22630-1 (PMC8113445; doi:10.1038/s41467-021-22630-1)
Supplement: Supplementary file 1 — Supplementary Information [file 41467_2021_22630_MOESM1_ESM.pdf]

Supplementary Information to Linking functional traits and demography to model species-rich communities

1 Supplementary Methods

1.1 Trait measurement protocol

For each sampled individual, we identified the species and measured seven functional traits. (i) Reproductive height is the distance between the highest reproductive organ and the ground; (ii) Vegetative height is the distance between the highest photosynthetic organ and the ground; (iii) specific leaf area (SLA) is the one-sided area of a fresh leaf divided by its oven-dry mass. (iv) Leaf dry matter content (LDMC) is the oven-dried mass of a leaf divided by its water-saturated fresh mass. It was measured using the partial rehydration method, which has been proven to give results similar to the full rehydration method [21]. (v) Leaf nitrogen concentration (LNC) is the total amount of nitrogen per unit of dry leaf mass. (vi) Leaf carbon concentration (LCC) is the total amount of carbon per unit of leaf dry mass. (vii) Leaf carbon isotopic ratio ($\delta^{13}C$). (vii) Leaf nitrogen isotopic ratio ($\delta^{15}N$).

To obtain reliable leaf trait estimates for SLA and LDMC, we collected a single non-senescent, non-grazed, non-frozen, well-developed leaf per individual [3]. For some individuals that had very small leaves and should thus lead to biased measures of area (ex. *Galium mollugo*), we selected a larger number of leaves. This was done case by case but consistently through the gradient. Non-photosynthetic tissue (e.g. petiole) was removed from the leaves before measurement. We measured leaf area using a portable laser leaf scanner (CID Bio-science Inc., Camas, WA, USA). We performed at least three measurements per sample to detect a potential scanner error.

For LNC, LCC, $\delta^{13}C$ and $\delta^{15}N$, dried and marble-ground leaf samples of 1–2 mg were analysed at the individual level with a continuous-flow isotope ratio mass spectrometer (Delta V Advantage; Thermo Scientific, Bremen, Germany) coupled to an elemental analyser (Flash EA1112; Thermo Scientific, Milan, Italy) at La Rochelle University-UMR CNRS LIENs.

1.2 Definition of functional trait axes used in the case study

We ran a Principal Component Analysis (R-package ade4 [4]) on the species-trait matrix and retained the three main orthogonal empirical functional trait axes to conduct our analysis. Missing values of a given functional trait were substituted by the mean of the trait across species. This appendix contains details about this analysis. We report the loadings of functional traits along the PCA axes (Supplementary Table 1), the relationship

Functional trait	Axis 1	Axis 2	Axis 3	Axis 4
Plant reproductive height (log)	-0.671	-0.277	0.601	0.036
Plant vegetative height (log)	-0.777	-0.396	0.344	- 0.037
SLA (log)	-0.612	0.387	-0.290	0.035
LDMC (log)	0.569	-0.546	0.315	0.112
Leaf carbon content (log)	0.245	-0.490	-0.363	0.699
Leaf nitrogen content (log)	-0.417	-0.245	-0.720	-0.222
Leaf $\delta^{13}C$	0.448	-0.417	-0.063	-0.729
Leaf $\delta^{15}N$	-0.408	-0.638	-0.328	-0.043
Explained variance	29.5%	19.5%	17.9%	13.6%

Supplementary Table 1: Scores of functional traits along the three first PCA axes

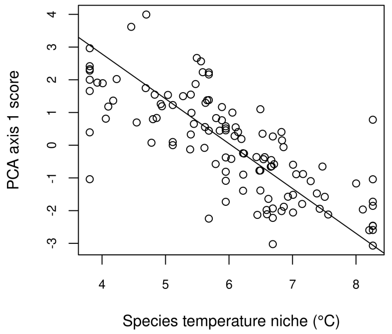

Supplementary Figure 1: Species mean thermal niche in relationship to species score along PCA axis 1 (Pearson's product-moment correlation two-sided test, $r = -0.746$ (%95 CI: [-0.817, -0.654]), $t = -12.073$, $df = 116$, $p < 2.2 \times 10^{-}16$)

between species score along the first axis and their "thermal niche", i.e. the average mean annual temperature of the plots where they were present (weighted by the number of sampled individuals) (Supplementary Figure 1) and the distribution of species scores across functional groups (Supplementary Figure 2).

1.3 Properties of the community model

1.3.1 Premises

The ordinary differential equation model is formulated as follows:

$$\frac{1}{B_{ij}}\frac{dB_{ij}}{dt} = g \times (\theta_j - \theta min_i) - c_i B_{ij} - l_i \sum_k B_{kj}$$

B_{ij} the biomass of species i in site j. θ_j the temperature in site j and g (constant across species), θmin_i, c_i, l_i demographic parameters of species i. g, l_i and c_i are all strictly positive for all species i.

1.3.2 Is the model globally stable?

There is an important literature about what how the behavior of Lotka-Volterra models is determined by the values or the structure of the interaction matrix coefficients [13],

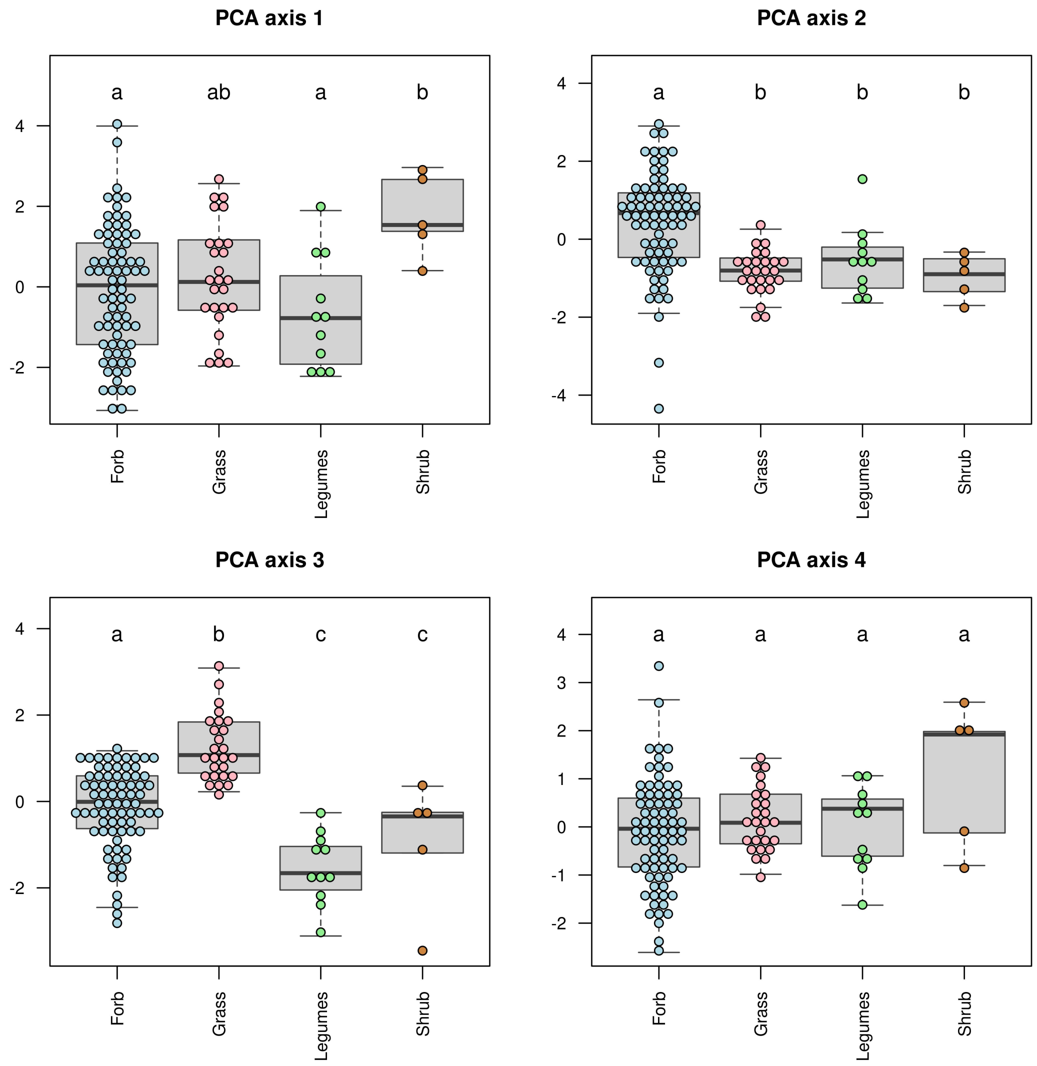

Supplementary Figure 2: PCA score axes across plant functional groups: forbs (76 species), grasses (26), and legumes (11) and shrubs (5). Boxplots indicate the median, first and third quartiles of each distribution. Whiskers represent the minimum and maximum values that remain inferior 1.5 times the interquartile range below or above the distribution median. Anova tests indicate that PCA scores were significantly different among functional groups for the first three axes (Axis 1: $F = 3.411$, $p = 0.020$; Axis 2: $F = 10.25$, $p = 5.0 \times 10^{-6}$; Axis 3: $F = 29.44$, $p = 3.6 \times 10^{-14}$; Axis 4: $F = 2.537$, $p = 0.06$). Letters above the boxplots symbolize the results of pairwise t-tests on demographic parameters among functional groups. Distinct letters characterize significantly different PCA score distributions among functional groups ($p \leq 0.05$ after adjustment by Holm's correction).

[14]. To our knowledge, the stability properties of the interaction matrix structure used in our study has not been studied, but we can demonstrate here that it always leads to a globally stable equilibrium.

The model can be reformulated as a classical Lotka-Volterra model:

$$\frac{1}{B_{ij}}\frac{dB_{ij}}{dt} = r_{ij} + \sum_{k} a_{ik} \times B_{kj}$$

$$r_{ij} = g \times (\theta_j - \theta min_i)$$

$$a_{ik} = \begin{cases} -(c_i + l_i) & \text{if } i = k \\ -l_i & \text{if } i \neq k \end{cases}$$

The matrice A with elements a_{ik} can be expressed with two diagonal matrices D and c and the matrix B containing only 1.

$$A = \begin{pmatrix} -c_1 & -l_1 & -l_1 & \cdots & -l_1 & -l_1 \\ -l_2 & -c_2 - l_2 & -l_2 & \cdots & -l_2 & -l_2 \\ -l_3 & -l_3 & -c_3 - l_3 & \cdots & -l_3 & -l_3 \\ \vdots & \vdots & \vdots & \ddots & \vdots & \vdots \\ -l_{n-1} & -l_{n-1} & -l_{n-1} & \cdots & -c_n - l_{n-1} & -l_{n-1} \\ -l_n & -l_n & -l_n & \cdots & -l_n & -c_n - l_n \end{pmatrix} = -DG = -D(J + C)$$

$$D = \begin{pmatrix} l_1 & & 0 \\ & \ddots & \\ 0 & & l_n \end{pmatrix} ; C = \begin{pmatrix} \frac{c_1}{l_1} & & 0 \\ & \ddots & \\ 0 & & \frac{c_n}{l_n} \end{pmatrix} ; J = \begin{pmatrix} 1 & \cdots & 1 \\ \vdots & \ddots & \vdots \\ 1 & \cdots & 1 \end{pmatrix}$$

If a diagonal matrix E exists such that $EA + A^T E$ is negative definite (i.e. the real part of its largest eigenvalue is negative), then the system is globally stable [9]. In our case, we can choose $E = D^{-1}$, then we get :

$$D^{-1}A + A^T D^{-1} = D^{-1}(-DG) + (-G^T D)D^{-1} = -G - G^T$$

Because G is symmetric, $-G - G^T = -2G$

This means that, if we can show that for any demographic parameter values, -G is negative definite (i.e. its largest eigenvalue is negative), then we know that the system is globally stable.

$-G = -J - C$. C is diagonal and J is constant 1. The eigenvalues of -C are its negative diagonal elements $-c_i/l_i$ and the smallest eigenvalue of J is 0 [11], thus the largest eigenvalue of $-J$ is also 0. The largest eigenvalue of the sum of two Hermitian matrices (J and C are symmetric) is smaller than the sum of the largest eigenvalues of the two matrices [12].

In our case, the largest eigenvalue of $-J - C$ is smaller than $max(-c_i/l_i) + 0 = -min(c_i/l_i)$. As c_i and l_i are strictly positive, that quantity is strictly negative. This means that $-2G$ is negative definite and that our community model is globally stable.

1.4 Properties of the transfer function

1.4.1 Why is g_i set constant?

Our model only fits the equilibrium of the ordinary differential equation model. The biomasses B^*_{ij} at equilibrium verify the following equations:

$$B^*_{ij} = 0$$

or

$$g_i \times (\theta_j - \theta min_i) - c_i B^*_{ij} - l_i \sum_k B^*_{kj} = 0$$

The second equation is equivalent to

$$(\theta_j - \theta min_i) - (c_i/g_i) B^*_{ij} - (l_i/g_i) \sum_k B^*_{kj} = 0$$

This indicates that the equilibrium would be left unchanged if we define new demographic parameters c'_i and l'_i as $c'_i = c_i/g_i$ $l'_i = l_i/g_i$ This shows that keeping g constant across species will not affect the estimation of the ODE equilibrium and would allow to explore the same parameter space with less parameters.

1.4.2 Why is b_c kept constant?

We use the notations of 2.2 and define the column vector $R_j = \{r_{ij}\}$ with the growth rates $r_{ij} = g_i \times (\theta_j - \theta_{min,i})$ of species i in site j.

The matrix A is defined by the demographic rate vectors $c = \{c_i\}$ and $l = \{l_i\}$ described in 2.2. The column vector of positive biomasses at equilibrium (B^*_j) is:

$$B^*_j = -A^{-1}R_j = (J + C)^{-1} D^{-1} R_j$$

And the total biomass in site j verify:

$$-uA^{-1}R_j = u(B + C)^{-1} D^{-1} R_j$$

with u the row vector $u = \{1, ..., 1\}$

The column vector of species relative abundances in site j is

$$\frac{A^{-1}R_j}{uA^{-1}R_j}$$

According our transfer function (Equation 2 in the main text),

$$l_i = exp(a_l \times E_{i,l} + b_l) = exp(a_l \times E_{i,l}) \times exp(b_l)$$

$$c_i = exp(c_l \times E_{i,c} + b_c) = exp(c_l \times E_{i,c}) \times exp(b_c)$$

We can demonstrate that when the model is calibrated with species relative abundances, the parameters b_c is redundant with b_l. Both parameters change the multiplying coefficient imposed on the demographic rates **c** and **l**. If those parameter values change then the new vectors of demographic rates c' and l' are $c' = x \times c$ and $l' = y \times l$ with x and y two scalars.

The new pairwise interaction matrix A' is:

$$A' = -yD \times (\frac{x}{y}B + C)$$

We can calculate the new column vector of relative abundances sites j as :

$$\frac{A'^{-1}R_j}{uA'^{-1}R_j} = \frac{(\frac{x}{y}B + C)^{-1}y^{-1}D^{-1}R_j}{u(\frac{x}{y}B + C)^{-1}y^{-1}D^{-1}R_j} = \frac{(\frac{x}{y}B + C)^{-1}D^{-1}R_j}{u(\frac{x}{y}B + C)^{-1}D^{-1}R_j}$$

With the new parametrization, the vector of relative species biomasses varies according to the ratio $\frac{x}{y}$, if those two scalars vary proportionally, then the relative species biomasses stay constant.

It is therefore sufficient to vary only b_c or b_l to explore the same parameter space.

1.4.3 Control parameters of the ODE

In our approach, we fit the equilibrium state of the ODE to empirical data. For a given set of parameters, the ODE thus has to run for a sufficient number of time steps to reach equilibrium and do so reasonably fast. Specifying the maximum number of time steps of the ODE is thus of paramount importance, given that a too small number of steps leads to an inaccurate estimate of the equilibrium state and a too large number of steps considerably slow down the MCMC. The running speed of the ODE depends further on the magnitude of the demographic parameter values, and thus also affects the speed of the calibration procedure. The values of parameters a_m and b_m control the mean and variance of each the demographic parameters, they were identified as critical features of the ODE behavior (time steps to convergence and running time). In contrast, the angles parameters ϕ_i affected only the ODE behavior through the correlation structure they define among demographic parameters. To control for this, we set boundaries on all parameters a_m and b_m despite them not being naturally bounded (6). We then tested the ODE behavior for each combination of (1) boundary values of a_m and b_m; (2) extreme correlation structure among demographic parameters (i.e. positive semi-definite correlation matrix with -1, 0 and 1 values); (3) at the smallest and largest temperature values θ_j. We visually control for the convergence of the 118 species and evaluate the running time of the ODE. This ultimately allowed us to fix a reasonable number of time steps for the ODE and adjust accordingly the prior distribution boundaries of the a_m and b_m parameters.

1.4.4 Properties of the hyperspherical parameterization

In our manuscript, we used the following formula to estimate demographic parameters:

$$\begin{aligned} E_{i,m} =& cos(\phi_{m,1}) \times t_{i,1} + \\ & \dots \\ & \prod_{k=1}^{n-1} sin(\phi_{m,k}) \times cos(\phi_{m,n}) \times t_{i,n} + \\ & \dots \\ & \prod_{k=1}^{N-1} sin(\phi_{m,k}) \times t_{i,N} \end{aligned}$$

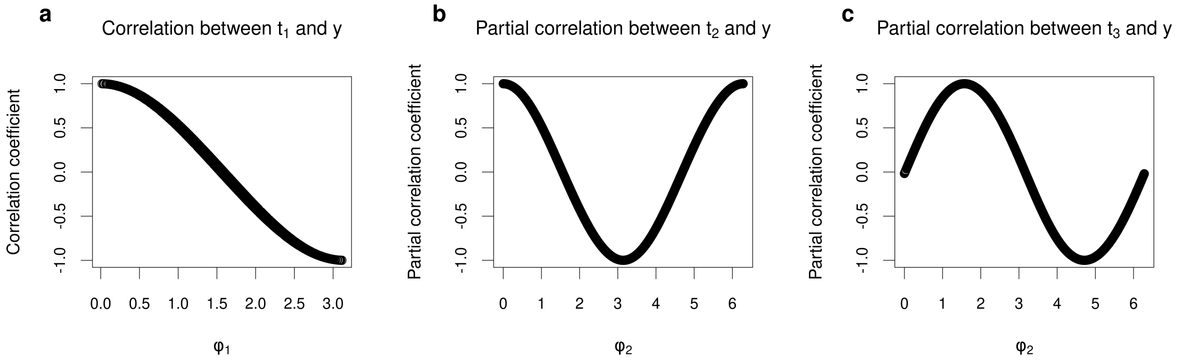

Supplementary Figure 3: (Partial) correlations between a response variable y and three trait vectors t_1, t_2 and t_3 as a function of angle parameters ϕ_1 and ϕ_2. The figure shows how the angle parameters allow the sampling of the correlation structure between y and t_1, t_2 and t_3.

$E_{i,m}$ is the link scale that links the unknown value of demographic parameter m of species i to the $t_{i,n}$ values of functional trait n of species i. This equation is general for any number N of functional traits. The N-1 angle parameters $\{\phi_{m,n}\}$ control the relationship between $E_{i,m}$ and $t_{i,n}$. $\phi_{i,n}$ vary within the range $[0, \pi]$ for $n < N - 1$ and $\phi_{i,N-1}$ varies within the range $[0, 2\pi]$.

Here we illustrate that the distribution of the angle parameters $\{\phi_{i,n}\}$ allows an efficient sampling of all possible correlations between the vector $D = \{d_{im}\}$ and the trait vectors contained in the matrix $T = \{t_{kj}\}$, for the case where N = 3. To do so, we generated three random vectors of 500 trait values t_1, t_2 and t_3 that follow a reduced and centered normal distribution. We then draw 2000 values of $\phi_{i,1}$, $\phi_{i,2}$ that follow a uniform law on the intervals $[0, \pi]$ and $[0, 2\pi]$ respectively. We then calculated a response vector y following this formula:

$$\begin{aligned} y_i =& cos(\phi_{i,1}) \times t_{i,1} + \\ & sin(\phi_1) \times cos(\phi_2) \times t_{i,2} + \\ & sin(\phi_1) \times sin(\phi_2) \times t_{i,3} \end{aligned} \tag{1}$$

We then calculated the correlation coefficient between y and t_1, the partial correlation coefficient between y and t_2 after controlling for t_1 and the partial correlation coefficient between y and t_3 after controlling for t_1. Results of the simulation are illustrated in Supplementary Figure 3.

1.4.5 Details about the prior and posterior distributions

The prior distributions of the posterior eleven transfer function parameters are indicated in Supplementary Table 2. The prior distributions of ϕ_1 and ϕ_2 were uninformative. We sampled $cos(\phi_1)$ in a uniform law in the range $[-1, 1]$, while ϕ_2 was sampled in a uniform law in the range $[0, 2\pi]$. We admit that a more intuitive prior distribution of ϕ_1 would be a uniform law between $[0, \pi]$. However, while those two prior distributions cover the same parameter space, the cosinus transformation of the prior distribution of ϕ_1 down-weights the sampling of strong correlations between demographic parameters and the first trait axis (Supplementary figure 4). Without the transformation, the correlations between

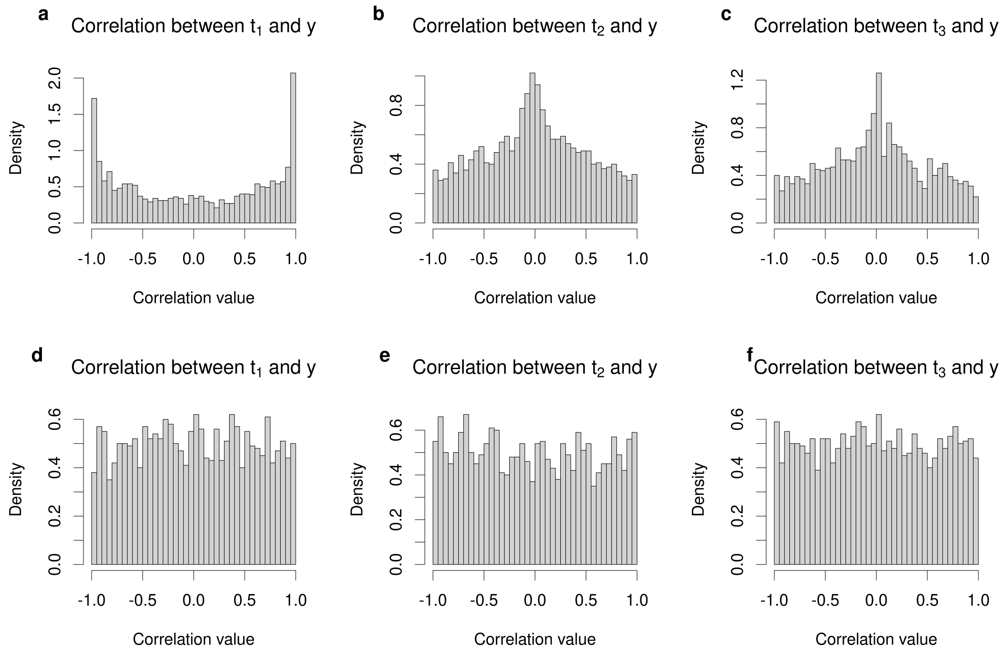

Supplementary Figure 4: Distribution of the correlation coefficients between the demographic parameter y and each of the three traits t_1 (**a**,**d**), t_2 (**b**,**e**), t_3 (**c**,**f**). y was calculated as a function of t_1, t_2 and t_3 as specified by Equation 1. t_1, t_2, t_3 were drawn independently in a normal law (200 values for each trait). In graphics **a-c**, 5000 values of parameter ϕ_1 were sampled in a uniform law between $[0, \pi]$ and 5000 values of parameter ϕ_2 is sampled in a uniform law between $[0, \pi]$. In graphics **d-f**, 5000 values of parameter $cos(\phi_1)$ were sampled in a uniform law between $[0, \pi]$ and 5000 values of parameter ϕ_2 is sampled in a uniform law between $[0, \pi]$.

the demographic parameters and the second and third trait axes tend to be smaller in absolute value than with the first axis.

We illustrate the posterior distribution of the transfer function parameters of the calibrated model in Supplementary Figure 5. The posterior distribution (red) is compared to the prior distribution of the transfer function parameters (blue). The full posterior distribution of the parameter is stored in the Source Data file.

2 Supplementary notes 1: robustness of the transfer function approach to the definition of trait axes

2.1 Does the posterior distribution change if trait axes are shuffled?

We tested if our approach is sensitive to the ordering of trait axes. When traits are shuffled, the set of ϕ_1 and ϕ_2 combination associated to each trait is modified by the new trait order (see Equation 1). For instance, with the original trait order, the coefficient that associates θ_{min} and the first trait axis (Supplementary Table 1) is $cos(\phi_1(\theta_{min}))$. But if that trait axis is set in third position, then the associated coefficient will be $sin(\phi_1(\theta_{min})) \times sin(\phi_2(\theta_{min}))$. If our methodology is robust to trait ordering, then we

Variable	Distribution	Range of the distribution
Minimum tolerated temperature (θ_{min})		
ϕ_1	$arcos(U(-1,1))$	$arccos([-1,1])$
ϕ_2	$U(0,2\pi)$	$[0,2\pi]$
a	$LogN(log(0.9),0.2)$	$[0.55,1.5]$
b	$N(-2.37,0.35)$	$[-3.0,-1.7]$
Sensitivity to competition (l)		
ϕ_1	$U(0,\pi)$	$[0,\pi]$
ϕ_2	$U(\pi,3\pi)$	$[\pi,3\pi]$
a	$Inv-Gamma(9,3.5)$	$[0.2,0.74]$
b	$N(-6.5,0.35)$	$[-7.1,-5.8]$
Intraspecific competition (c)		
ϕ_1	$U(0,\pi)$	$[0,\pi]$
ϕ_2	$U(\pi,3\pi)$	$[\pi,3\pi]$
a	$Inv-Gamma(40,46)$	$[0.8,1.50]$
b	/	fixed (b = -3.8)

Supplementary Table 2: Prior distribution of the transfer function parameters for the three traits model.

Variable	Distribution	Range of the distribution
Minimum tolerated temperature (θ_{min})		
ϕ_1	$arcos(N(-0.95,0.4))$	$arccos([-1,1])$
ϕ_2	$arcos(U(-1,1))$	$arccos([-1,1])$
ϕ_3	$U(0,2\pi)$	$[0,2\pi]$
a	$LogN(log(0.8),0.01)$	$[0.55,1.5]$
b	$N(-2.37,0.01)$	$[-3,-1.7]$
Sensitivity to competition (l)		
ϕ_1	$arcos(N(0.95,0.4))$	$arccos([-1,1])$
ϕ_2	$arcos(U(-1,1))$	$arccos([-1,1])$
ϕ_3	$U(0,2\pi)$	$[0,2\pi]$
a	$Inv-Gamma(40,12.3)$	$[0.2,0.72]$
b	$N(-6.1,0.2)$	$[-7.1,-5.5]$
Intraspecific competition (c)		
ϕ_1	$arcos(N(0.58,0.3))$	$arccos([-1,1])$
ϕ_2	$arcos(N(0.98,0.4))$	$arccos([-1,1])$
ϕ_3	$U(0,2\pi)$	$[0,2\pi]$
a	$Inv-Gamma(40,46)$	$[0.8,1.50]$
b	/	fixed (b = -3.8)

Supplementary Table 3: Prior distribution of the transfer function parameters for the four traits model.

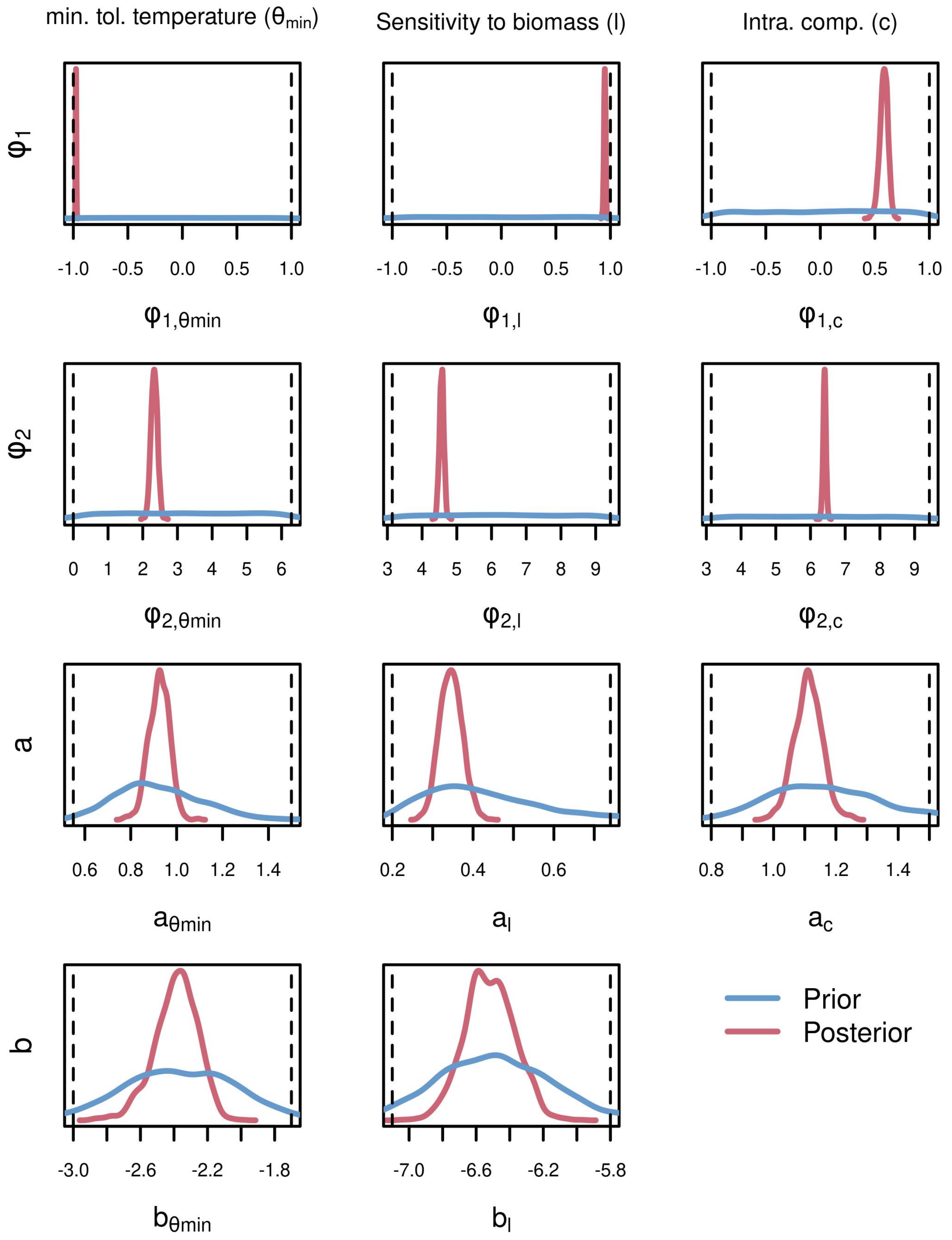

Supplementary Figure 5: Posterior distribution of the transfer function parameters. Red curves indicate the posterior distribution. Blue curves indicate the prior distribution and the dashed lines, the set limits of the prior distribution.

expect if the trait order is changed that:

- the a and b parameters to be similar
- the ϕ_1 and ϕ_2 parameters to be different...
- ... but the coefficient associated to each trait axis to be similar.

To test this, we ran the calibration procedure for all possible ordering of the three trait axes. For each calibration procedure with alternative trait ordering, we ran three chains. We then reported the coefficient value associated to each trait axis and of the a and b parameters, the posterior likelihood of the model, its convergence, the DIC and the pseudo R^2 (Supplementary Table 4. The Deviance Information Criterion was calculated following Gelman et al. (2004)[7]. Note that the effective number of parameters was calculated as half of the variance of the deviance function across the posterior, as this estimation was more robust to different trait order than the formulation of Spiegelhalter et al. (2002)[18].

We found out that trait order did not modify the posterior distribution of the model. However, the calibration procedure needed, for some trait orderings, a larger number of burning steps to estimate the posterior compared to the original trait ordering.

2.2 Does the posterior distribution change if we add a fourth trait axis?

We further tested if the posterior distribution of the model would be strongly changed by the addition of the fourth trait axis (Supplementary Table 4). The increased number of parameters required us to constrain further the priors of the parameters compared to the three traits model. Indeed, some parameters, in particular b_l and $b_{\theta min}$ on one hand, were partially non identifiable to each other: the MCMC chains tended to drift towards a simultaneous decrease of $b_{\theta min}$ and increase of b_l based on a minimal likelihood variation. That drift was not biological credible as a very low value of $b_{\theta min}$ indicated that all species are potentially able to have a positive biomass everywhere on the temperature gradient. To prevent that drift, we impose a constrained prior on $b_{\theta min}$ with a much reduced standard deviation compared to the original model. Similarly, a_l and $a_{\theta min}$ were also partially non identifiable, we thus impose a constrained prior on $a_{\theta min}$. Based on successive runs of the calibration with different priors, we further adjusted the parametrization of the priors of b_l, a_l and a_c to help the calibration of the correlation parameters.

Finally we set weakly informative priors on the correlation parameters $\phi_{1,\theta min}$, $\phi_{1,l}$, $\phi_{1,c}$, $\phi_{2,c}$. Those priors were defined as truncated normal distributions, their mean was defined based on the model calibrated with three traits. In short, that scheme assumes that the strong correlations calibrated with the three traits model are likely to be maintained to an extent in the four traits model. Those correlations were the strong negative correlation between θmin and the PCA axis 1, the strong positive correlation between l and the PCA axis 1, the moderately strong positive correlation between c and the PCA axis 1 and the moderately strong positive correlation between c and the PCA axis 2 (Supplementary Tables 1, 4). The other correlation parameters had uninformative priors as there were no sufficient evidences from the three traits model to constrain their distribution.

Despite the restriction imposed on the priors, the chains did not converge after 100 000 steps. We thus only display in Supplementary Table 4, the posterior distribution

of three of the eight chains that converged to the highest likelihood value. The chains notably had trouble converging to the same value phi_3 parameters that control the partial correlation to the third and fourth axis after factoring out the correlation to the first two. This behavior may be due to the lack of signal on those two axes. The model was significantly improved by the addition of a fourth axis (DIC = 6366.8 against 6476.4 with three traits) but that improvement was limited: even the chains that converged to the highest likelihood value only had a slightly higher pseudo R^2: 0.62 against 0.59 for the model calibrated with three traits. The inclusion of a fourth trait axis also did not change radically the correlation structure between traits and demographic parameters.

3 Supplementary notes 2: comparative analyses

We performed additional analyses to compare the analysis approach proposed in this study (LV method with transfer function) with alternative, established analyses frameworks that use community data, possibly in connection with trait data. As candidates for such analyses, we identified species distribution models (SDMs), joint species distribution models (jSDMs), and fourth corner analysis. We applied these three methods to our data, and compare the results in terms of model fit, but also information provided and ecological conclusions.

3.1 Species distribution models and joint species distribution models

Species distribution models (SDM) describe species occurrences as a function of environmental covariates [6]. One shortcoming of SDM is that they disregard species interactions and assume that species occurrences depend only on the environment and space. jSDM, a novel type of model, extend SDM by accounting for unobserved species interactions with species-species associations [16].

SDM: the following equation describes the function of the environmental covariate (temperature) as an orthogonal polynomial of degree 2 on the link scale:

$$\mu_{ji} = \beta_{0i} + \beta_{1i} * X_j + \beta_{2i} * X_j^2$$

We apply the inverse multinomial logit link (also called softmax) over the sites j get a vector of probabilities for each site

$$\mathbf{z}_j = \frac{\exp(y_{ji})}{\sum_{i=1}^{I} \exp(\mu_{ji})}$$

and calculate the multinomial likelihood:

$$y_j \sim Multinomial(\mathbf{z}_j)$$

The model was fit using the R-package RStan [19] with 2 chains, 50,000 burn-in and 50,000 MCMC sampling steps. We set normal priors for the parameters:

$$\beta_0, \beta \sim N(0.0, 3.0)$$

Trait ordering	1-2-3	1-3-2	2-1-3	2-3-1	3-1-2	3-2-1	1-2-3-4
Minimum tolerated temperature (θ_{min})							
Trait 1 coefficient	-0.976	-0.976	-0.976	-0.976	-0.976	-0.976	-0.945
Trait 2 coefficient	-0.151	-0.153	-0.148	-0.149	-0.149	-0.150	-0.213
Trait 3 coefficient	0.159	0.157	0.156	0.159	0.157	0.157	0.146
Trait 4 coefficient	/	/	/	/	/	/	0.201
$a_{\theta min}$	0.926	0.926	0.927	0.927	0.925	0.927	0.801
$b_{\theta min}$	-2.380	-2.360	-2.400	-2.400	-2.390	-2.390	-2.370
Sensitivity to competition (l)							
Trait 1 coefficient	0.948	0.950	0.950	0.949	0.949	0.949	0.935
Trait 2 coefficient	-0.047	-0.045	-0.047	-0.046	-0.046	-0.045	0.108
Trait 3 coefficient	-0.314	-0.310	-0.309	-0.311	-0.311	-0.312	-0.281
Trait 4 coefficient	/	/	/	/	/	/	- 0.188
a_l	0.345	0.347	0.343	0.342	0.346	0.345	0.294
b_l	-6.520	-6.520	-6.500	-6.490	-6.520	-6.510	-6.050
Intraspecific competition (c)							
Trait 1 coefficient	0.584	0.588	0.582	0.581	0.581	0.583	0.607
Trait 2 coefficient	0.805	0.803	0.807	0.807	0.808	0.806	0.733
Trait 3 coefficient	0.100	0.097	0.101	0.102	0.101	0.099	0.102
Trait 4 coefficient	/	/	/	/	/	/	-0.288
a_c	1.110	1.120	1.110	1.110	1.110	1.110	1.180
Model characteristics							
Steps per chain	50000	120000	50000	100000	50000	50000	90000
Burning steps	35000	95000	35000	85000	35000	35000	75000
Number of chains	8	3	3	3	3	3	3
Gelman msrf	1.01	1.07	1.02	1.05	1.01	1.01	1.03
Log-likelihood	-3232.6	-3232.6	-3232.8	-3232.7	-3232.8	-3232.6	-3175.5
DIC	6476.5	6476.0	6476.9	6476.8	6476.8	6476.5	6366.6
Pseudo R^2	0.59	0.59	0.59	0.59	0.59	0.59	0.62

Supplementary Table 4: Characteristics of the calibrated models when the traits are shuffled (first six columns on the left) or if the fourth PCA trait axis is added (seventh column). Trait coefficients refer to the value of the combination of ϕ_1 and ϕ_2 (and eventually ϕ_3) parameter values as specified by Equation 1. Trait coefficients and a and b parameter values refer to their respective median value across the posterior. Gelman msrf refers to Gelman's multivariate scale reduction factor, DIC refers to the Deviance Information Criterion[7]

sjSDM MLE: the originally proposed jSDM model is based on the multivariate probit model [16] which extends SDMs by accounting for species-species associations with a multivariate normal distribution:

$$\mu_{ji} = \beta_{0i} + \beta_{1i} * X_j + \beta_{2i} * X_j^2 + e_{ji}$$
$$e_j \sim MVN(0, \Sigma)$$

sjSDM is a new method to estimate jSDMs that is based on a Monte-Carlo approximation of the multivariate probit model [15]. The approximation allows us to forward different probability distributions through the Monte-Carlo approximation to estimate the species-species associations. We extended the sjSDM R-package by a multinomial likelihood, similar to the SDM (previous section) with an inverse multinomial logit link. The implementation is available in the "multinomial" branch of sjSDM: `https://github.com/TheoreticalEcology/s-jSDM/tree/multinomial`. The sjSDM model was fit in 600 iterations, 4000 Monte-Carlo samples for the approximation, and a step size of one. The species-species association matrix was parametrized by a $118*6$ matrix. The Monte-Carlo approximation leads to a variant of the multinomial distribution, thus we calculated post-hoc the multinomial likelihood with the predictions of sjSDM to compare it to the other models.

sjSDM MCMC: the sjSDM model was also estimated via MCMC sampling. We used a Differential-Evolution MCMC sampler (Bayesian Tools package) as for the Transfer function approach described in the main manuscript. We recorded the posterior for 100,000 sampling steps after 10,000,000 burn-in steps. We set normal priors for all parameters:

$$\beta_0, \beta \sim N(0.0, 3.0)$$

jSDM - latent variable model (LVM): the LVM is another method to estimate jSDMs that was introduced to reduce the total number of parameters to be estimated in a jSDM [22]. The LVM introduces k latent environmental covariates (η, normally 2 - 8) and species respond to these artificial environmental covariates via their factor loadings (λ). The species are connected by their factor loadings:

$$\mu_{ji} = \beta_{0i} + \beta_{1i} * X_j + \beta_{2i} * X_j^2 + \sum_{k=1}^{K} \eta_{ik} * \lambda_{ki}$$

From here, the multinomial likelihood is calculated as for the SDM. The factors loadings can then be used to construct the species-species association matrix:

$$\Sigma = \lambda^T * \lambda$$

The model was fit using the R-package RStan [19] with 2 chains, 2 latent variables, 50,000 burn-in and 50,000 MCMC sampling steps. We set normal priors for all parameters:

$$\beta_0, \beta, \eta, \lambda \sim N(0.0, 3.0)$$

Saturated model: we compared the SDM and jSDM models with a saturated SDM model (same link function and likelihood as for the SDM). For that, we used a model with as many parameter as there are sites, which means $16 * 118$ ($sites * species$) parameters. The model was fit using the R-package RStan [19] with 2 chains, 2 latent variables, 50,000 burn-in and 50,000 MCMC sampling steps. We set normal priors for all parameters:

$$\beta \sim N(0.0, 3.0)$$

Comparison methodology First, we evaluated the performance of the SDM and the jSDMs using Nagelkerke's pseudo R^2, DIC and AIC. We further estimated the ROC curves for each model as well as the related AUC score by analyzing a complementary dataset collected on the same plots. Alongside the transect data, (more) exhaustive presence/absence botanical data were collected. This allowed us to calculate the ability of the modeling approaches to predict 'true' presence/absence with AUC scores (area under the curve) and to penalize approaches that assume that a species not sampled along a transect was truly absent from the studied plot[8]. ROC curves and AUC scores were computed using the R-package *pROC* [17].

Second, we compared species abundances predictions among modeling approaches. To do so, we quantified the correlation between the log-transformed relative abundances of each model to the log-transformed relative abundances predicted by the saturated model, here used as a reference. Those correlations were calculated by adding species incrementally: first, including only the most sampled species (in total number of sampled individuals), then the two most sampled species etc... until all 118 species were included.

Comparison to the transfer function approach The SDM and jSDMs fit better the data than the transfer function approach (see Table S5). Pseudo R^2 vary between 0.760 and 0.961 against 0.590 for the transfer function approach. The SDM and the JSDM-LVM approaches have a lower AIC (3840 and 3183 against 6477). The jSDMs fitted without latent variables are however less parsimonious than the transfer function approach and have a higher AIC (7127 and 7439) than the transfer function approach which is due to their high number of parameters (1062 against 11). The transfer function approach has an AUC score (0.68) that is only slightly inferior to the SDM and jSDM approaches ($0.68 < \text{AUC} < 0.79$) showing that it has a comparable ability to predict species presence/absence (see Figure S6). This close performance likely reflects how the modeling approaches treat species absence along transects. SDM and jSDM approaches treat those absences as a sign of a species absence, while the transfer function approach may still model those species as present if it has the right functional trait values.

When only including well sampled species, the different modeling approaches closely match in their prediction of species relative abundances (see Figure S7). For instance, if we only include the 30 most sampled species (i.e. sampled more than twenty times across plots, vertical dashed line on Figure S7), the predicted relative abundances are well correlated to the relative abundances predicted by the saturated model ($r > 0.44$). This matching degrades when species sampled only a few times are included: when all species are included, the transfer function approach matches less well the saturated model ($r = 0.28$) compared to the other modeling approaches ($r > 0.47$). To summarize, the transfer function approach and the SDM/jSDM approaches model well-sampled species similarly but diverge in their modeling of species with a lower sampling coverage.

3.2 Fourth corner method

The fourth-corner method describes trait-environment associations [5]. To do so, it combines the table containing environmental measurements (in our case, one variable) across sites (in our case, sixteen sites), the table containing species abundances across sites, and the table containing trait measurements across species (in our case, 8 traits across 118 species). The analysis provides a measurement of the strength of the association (Pearson's correlation) tested against a combination of two null models that randomizes

Approach	Log-Lik.	AIC	DIC	Pseudo R2	Parameters	AUC
Transfer function	-3227	6476	6477	0.590	11	0.68
SDM	-1690	4095	3840	0.935	354	0.73
sjSDM (MLE)	-2523	7128	/	0.819	1062	0.68
sjSDM (MCMC)	-2766	7439	13269	0.760	1062	0.70
jSDM-LVM	-1317	3897	3183	0.961	626	0.79
Saturated model	-923	/	/	0.979	2124	0.73

Supplementary Table 5: Comparison of the transfer function with species distribution models approaches. We reported the fit of the model (log-Likelihood and Nagelkerke's pseudo R^2), metrics of model quality (AIC and DIC) and the number of estimated parameters. The area under the curve index (AUC) indicates the ability of the model to predict the presence and absence of species in plots based on exhaustive botanical releves.

Functional trait	Pearson's r	P-value	P-value (adjusted)
Plant reproductive height (log)	0.585	0.001	0.008
Plant vegetative height (log)	0.608	0.001	0.008
SLA (log)	0.216	0.066	0.264
LDMC (log)	-0.172	0.181	0.543
Leaf carbon content (log)	-0.0421	0.728	1.000
Leaf nitrogen content (log)	-0.0353	0.763	1.000
Leaf $\delta^{13}C$	-0.379	0.002	0.012
Leaf $\delta^{15}N$	0.279	0.016	0.080

Supplementary Table 6: Fourth-corner statistic between plant functional traits and mean annual temperature. It was calculated through Pearson's r. Significance was established with a two-sided test through a permutation of both sites and species. P-values were further adjusted for multiple testing with the Holm correction [10].

either sites or species [20]. The analysis was run using the R-package *ade4* [4]

Comparison to the transfer function approach The fourth corner analysis indicates that plant reproductive and vegetative height increase with mean annual temperature (r = 0.59, p = 0.001; r = 0.61, p = 0.001) while leaf $\delta^{13}C$ diminishes (r = -0.37, p = 0.01). Leaf $\delta^{15}N$ marginally increases as well (r = 0.28, p = 0.08). The other traits did not have a significant connection to mean annual temperature (Table S6). The traits highlighted by the fourth corner analysis correspond to the main demographic trade-off calibrated by the transfer function approach (see Figure 2). Minimum tolerated temperature and Sensitivity to biomass (and to a lesser extent intraspecific competition) were also calibrated to correlate mainly with height traits and leaf isotopic traits. Those demographic parameters control strongly the species turnover modeled by this community model [2].

These results shows how both our approach and the fourth corner approach retrieve similar trait-environment relationships that are structuring community turnover. However they do so through different means: the fourth corner does so through the analysis of species abundances along environmental gradient while our approach does so through a community model that explicitly includes hypothetical demographic processes.

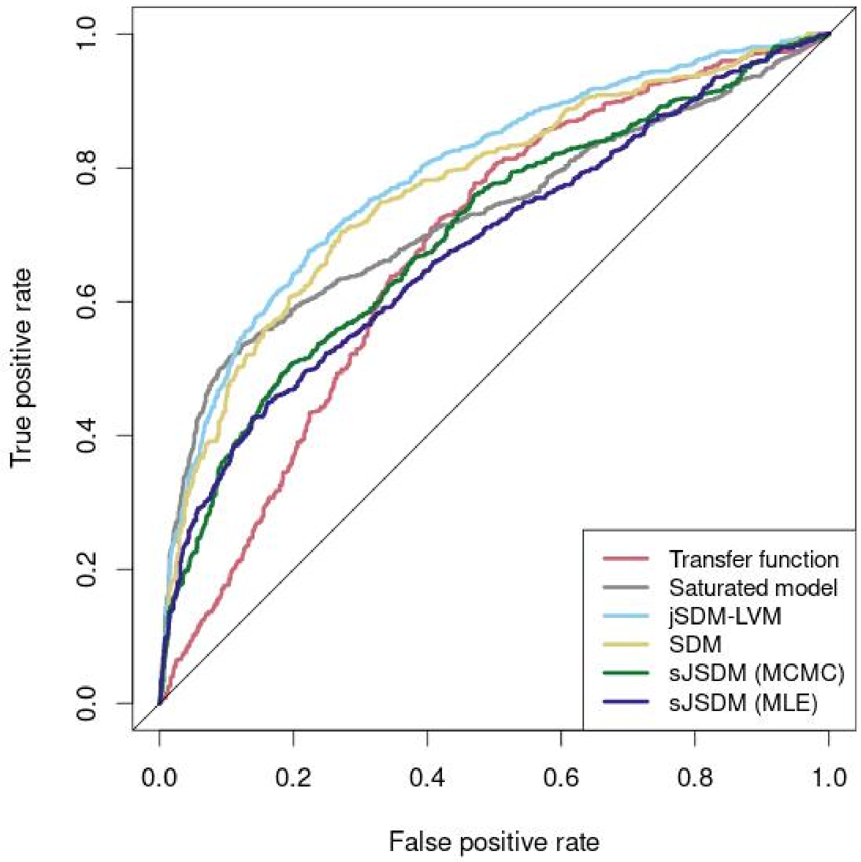

Supplementary Figure 6: ROC 'receiver operating characteristic' curves for the transfer function approach (pink), the species distribution model (yellow), the joined species distribution models (green and blues) and the saturated model (grey).

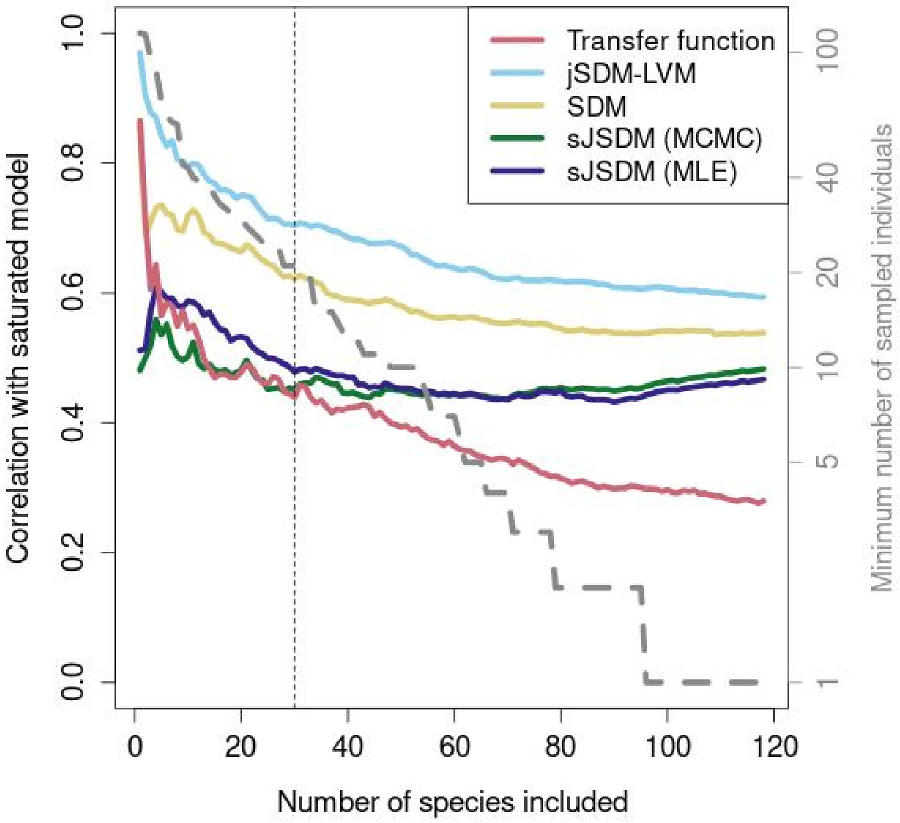

Supplementary Figure 7: Matching of predicted relative abundances between approaches as a function of the number of species included. Here we measured the correlation between the log-transformed relative abundances of each modeling framework (colored full lines) and the log-transformed relative abundances predicted by the saturated model. Those correlations were calculated by adding species incrementally (x-axis): first, including only the most sampled species (in number of sampled individuals), then the two most sampled species etc... until all 118 species were included. The gray dashed line and right-side y-axis indicate the corresponding minimal number of sampled individuals per species

Supplementary References

[1] Braun-Blanquet, J. *Über den Deckungswert der Arten in den Pflanzengesellschaften der Ordnung Vaccinio-Piceetalia.* Bischofberger & Company, 1946).

[2] Chalmandrier, L., Albouy, C. & Pellissier L. Species pool distribution along functional trade-offs shape plant productivity-diversity relationships. *Scientific Reports*, **7 (1)**, 1-11. (2017)

[3] Cornelissen, J.H.C., Lavorel, S., Garnier, E., Diaz, S., Buchmann, N., Gurvich, D.E., et al. A handbook of protocols for standardised and easy measurement of plant functional traits worldwide. *Australian Journal of Botany*, **51**, 335–380. (2003)

[4] Thioulouse, J., Dray, S., Dufour, A.-B., Siberchicot, A., Jombart, T. & Pavoine, S. Multivariate Analysis of Ecological Data with ade4. Springer New York, New York, NY.978-1-4939-8850-1 (2018)

[5] Dray, S. & Legendre, P. Testing the species traits-environment relationships: the fourth-corner problem revisited. *Ecology*, **89**, 3400-3412. (2008)

[6] Elith, J., & Leathwick, J. R. Species distribution models: ecological explanation and prediction across space and time. *Annual review of ecology, evolution, and systematics*, **40**, 677-697. (2009)

[7] Gelman, A., Carlin, J. B., Stern, H. S., Rubin, D. B.. Bayesian Data Analysis: Second Edition. Texts in Statistical Science, 2004. CRC Press.

[8] Godínez-Alvarez, H., Herrick, J. E., Mattocks, M., Toledo, D. & Van Zee, J. Comparison of three vegetation monitoring methods: their relative utility for ecological assessment and monitoring. *Ecol. Indic.* **9**, 1001–1008 (2009).

[9] Hofbauer, K. & Siegmund, K. Stability of n-species communities. In J. Hofbauer & K. Sigmunds (Eds.), *Evolutionary Games and Population Dynamics* (pp. 191-202). Cambridge: Cambridge University Press, 1998.

[10] Holm, S. A simple sequentially rejective multiple test procedure. *Scandinavian Journal of Statistics* **6**, 65–70 (1979).

[11] Horn, R.A. & Johnson, C.R. 0.2.8 The all-ones matrix and vector. Matrix Analysis. Cambridge University Press, 2012. ISBN 9780521839402.

[12] Horn, R.A. & Johnson, C.R. Eigenvalue inequalities for Hermitian matrices. Matrix Analysis. Cambridge University Press, 2012. ISBN 9780521839402.

[13] Logofet D. Stronger-than-Lyapunov notions of matrix stability, or how "flowers" help solve problems in mathematical ecology. Linear algebra and its applications 398: 75-100. (2005)

[14] May, R.M. Stability and complexity in model ecosystems. Princeton University Press. (1973)

[15] Pichler, M., & Hartig, F. A new method for faster and more accurate inference of species associations from novel community data. *arXiv preprint arXiv* :2003.05331. (2020)

[16] Pollock, L.J., Tingley, R., Morris, W.K., Golding, N., O'Hara, R.B., Parris, K.M., *et al.* Understanding co-occurrence by modelling species simultaneously with a Joint Species Distribution Model (JSDM). *Methods in Ecology and Evolution*, **5**, 397–406. (2014)

[17] Robin, X., Turck, N., Hainard, A., Tiberti, N., Lisacek, F., Sanchez, J.-C., Müller, M. pROC: an open-source package for R and S+ to analyze and compare ROC curves. *BMC Bioinformatics*, **12**, 77. (2009) `https://doi.org/10.1186/1471-2105-12-77`

[18] Spiegelhalter, D. J., Best, N. G., Carlin, B. P., van der Linde, A. Bayesian measures of model complexity and fit. *Journal of the Royal Statistical Society, Series B.* 64 (4): 583–639. (2002)

[19] Team, Stan Development. RStan: the R interface to Stan. *R package version 2.21.2.* (2020)

[20] ter Braak, C., Cormont, A., and Dray, S. Improved testing of species traits-environment relationships in the fourth corner problem. *Ecology* 93: 1525-1526. (2002)

[21] Vaieretti, M.V., Díaz, S., Vile, D. & Garnier, E. Two measurement methods of leaf dry matter content produce similar results in a broad range of species. *Annals of botany*, **99**, 955–958 (2007).

[22] Warton, D. I., Blanchet, F. G., O'Hara, R. B., Ovaskainen, O., Taskinen, S., Walker, S. C., & Hui, F. K. So many variables: joint modeling in community ecology. *Trends in Ecology & Evolution*, **30**(12), 766-779. (2015)
