# Supplementary material for: Linking functional traits and demography to model species-rich communities
Source: Nat Commun. 2021 May 11;12:2724. doi: 10.1038/s41467-021-22630-1 (PMC8113445; doi:10.1038/s41467-021-22630-1)
Supplement: Supplementary file 3 — Reporting Summary [file 41467_2021_22630_MOESM3_ESM.pdf]

nature research

Corresponding author(s): Loic Chalmandrier, Loic Pellissier

Last updated by author(s): Feb 26, 2021

Reporting Summary

Nature Research wishes to improve the reproducibility of the work that we publish. This form provides structure for consistency and transparency in reporting. For further information on Nature Research policies, see our Editorial Policies and the Editorial Policy Checklist.

Statistics

For all statistical analyses, confirm that the following items are present in the figure legend, table legend, main text, or Methods section.

n/a	Confirmed	
☐	☒	The exact sample size (*n*) for each experimental group/condition, given as a discrete number and unit of measurement
☐	☒	A statement on whether measurements were taken from distinct samples or whether the same sample was measured repeatedly
☐	☒	The statistical test(s) used AND whether they are one- or two-sided<br>*Only common tests should be described solely by name; describe more complex techniques in the Methods section.*
☐	☒	A description of all covariates tested
☐	☒	A description of any assumptions or corrections, such as tests of normality and adjustment for multiple comparisons
☐	☒	A full description of the statistical parameters including central tendency (e.g. means) or other basic estimates (e.g. regression coefficient) AND variation (e.g. standard deviation) or associated estimates of uncertainty (e.g. confidence intervals)
☐	☒	For null hypothesis testing, the test statistic (e.g. *F*, *t*, *r*) with confidence intervals, effect sizes, degrees of freedom and *P* value noted<br>*Give P values as exact values whenever suitable.*
☐	☒	For Bayesian analysis, information on the choice of priors and Markov chain Monte Carlo settings
☒	☐	For hierarchical and complex designs, identification of the appropriate level for tests and full reporting of outcomes
☐	☒	Estimates of effect sizes (e.g. Cohen's *d*, Pearson's *r*), indicating how they were calculated

Our web collection on statistics for biologists contains articles on many of the points above.

Software and code

Policy information about availability of computer code

Data collection	We used R 3.5.3 and R-packages ade4 1.7.16 and BayesianTools 0.1.7. This is specified in the main text of the manuscript
Data analysis	The data are now stored on a Github archive.

For manuscripts utilizing custom algorithms or software that are central to the research but not yet described in published literature, software must be made available to editors and reviewers. We strongly encourage code deposition in a community repository (e.g. GitHub). See the Nature Research guidelines for submitting code & software for further information.

Data

Policy information about availability of data

All manuscripts must include a data availability statement. This statement should provide the following information, where applicable:

- Accession codes, unique identifiers, or web links for publicly available datasets
- A list of figures that have associated raw data
- A description of any restrictions on data availability

Data are archived in the code repository DOI: 10.5281/zenodo.4556425. Source data are provided with this paper.

Field-specific reporting

Ecological, evolutionary & environmental sciences study design

All studies must disclose on these points even when the disclosure is negative.

Study description	The data used in this study already led the following article: Chalmandrier et al. 2017 'Spatial scale and intraspecific trati variability mediate assembly rules in alpine grasslands' - Journal of Ecology.<br>The study was conducted in the central French Alps. Subalpine grasslands dominated the bottom of the gradient while sparsely vegetated alpine meadows characterized higher elevations. The ten sites were evenly distributed along the elevation gradient (on average 100-m elevation difference apart from each other and separated by a geographical distance of 340 m on average) on the same south-facing slope. In each site, we placed two non-overlapping plots of 10 by 10 m a few meters apart in an homogeneous vegetation.
Research sample	The research sample was a vegetation plot of 10m by 10 m where 110 plant individuals were sampled. On each individual, eight functional traits were measured. The sample is meant to reflect the compositional and functional diversity of the local vegetation community<br>In each plot, a botanical survey was made to get a more complete list of present species.
Sampling strategy	In each quadratic plot, we sampled plant individuals along two transects that followed its diagonals. 110 individuals were sampled for functional traits in each plot. The number of individuals was constrained by available resources and was judged sufficient to evaluate the local functional diversity.
Data collection	In each quadratic plot, we sampled plant individuals as exposed above. The data collectors were the authors of the article Chalmandrier et al. 2017. Plots were sampled in July 2012 during the peak of vegetation productivity over three weeks. Exact days are not available anymore. Each plot was sampled once because it was constrained by available resources and time.
Timing and spatial scale	Each plot was sampled once in July 2012 over three weeks. Exact days are not available anymore. Sampling was made in July 2012 at the peak of vegetation productivity over three weeks (exact days are not available anymore). Plant individuals were sampled along two transects in a 10 by 10 m plots. Each plot was sampled once because it was constrained by available resources and time.
Data exclusions	Compared to the original study, we excluded one site at the lowest elevation. The one at the bottom of the gradient was excluded because of peculiar environmental conditions (closer to a stream and below the treeline in a wood clearing). The botanical composition of those communities made them outliers compared to the progressive vegetation turnover displayed by the other sites. In consequence, the mechanisms described by our community model were insufficient to describe the local community structure.
Reproducibility	Each site was geolocalised and plots were permanently marked with small wooden poles (~10cm above ground leve) that were left after the field work to mark the corner of each plot. This ensures that plots could be found again and the study replicated.
Randomization	There is no experimental treatment that would require a randomization of the samples.
Blinding	Transect lines were marked before being set in the vegetation. At each mark, the plant individual touching the mark was sampled. This ensures that sampled individuals are chosen at random and regardless of the field work participants prior expectations about the system.

Did the study involve field work? ☒ Yes ☐ No

Field work, collection and transport

Field conditions	Sampling was made in July 2012 at the peak of vegetation productivity. Based on the SAFRAN meteorological model developed by Meteo France for the French Alps (Durand et al. 2009 - Journal of Applied Meteorology and Climatology). Mean annual air temperature varied between 3.8°C and 0.8°C and annual precipitations between 919 and 1186 mm.<br><br>Durand, Y., Laternser, M., Giraud, G., Etchevers, P., Lesaffre, B. & Merindol, L. (2009) Reanalysis of 44 yr of climate in the French Alps (1958–2002): methodology, model validation, climatology, and trends for air temperature and precipitation. Journal of Applied Meteorology and Climatology, 48, 429–449.
Location	Data collection was conducted in the central French Alps (45.12°N, 6.40°E). Ten sites were studied along a continuous 775m elevation gradient (1850 - 2625m) in a cow grazed pasture.
Access & import/export	The cow pasture is easily accessible from the main road and through hiking trails. The installation (exclosure to prevent cow grazing) and plant sampling were allowed by the manager of the pasture unit.
Disturbance	Access on the pasture was made on foot, rather than vehicules to avoid damaging the vegetation. No significant disturbance was caused by the data collection apart from the sampling of plant individuals. Small wooden poles (~10cm above ground leve) were left after the field work to mark the corner of each plot. Any damage on the vegetation was not long-lasting as after field work, the pasture was and is still used as cow pasture ground. After the exclosures were removed and cows proceed to graze the sampled vegetation.

Reporting for specific materials, systems and methods

We require information from authors about some types of materials, experimental systems and methods used in many studies. Here, indicate whether each material, system or method listed is relevant to your study. If you are not sure if a list item applies to your research, read the appropriate section before selecting a response.

Materials & experimental systems

n/a	Involved in the study
☒	☐ Antibodies
☒	☐ Eukaryotic cell lines
☒	☐ Palaeontology and archaeology
☒	☐ Animals and other organisms
☒	☐ Human research participants
☒	☐ Clinical data
☒	☐ Dual use research of concern

Methods

n/a	Involved in the study
☒	☐ ChIP-seq
☒	☐ Flow cytometry
☒	☐ MRI-based neuroimaging
