# Supplementary material for: Linking functional traits and demography to model species-rich communities
Source: Nat Commun. 2021 May 11;12:2724. doi: 10.1038/s41467-021-22630-1 (PMC8113445; doi:10.1038/s41467-021-22630-1)
Supplement: Supplementary file 4 — Source Data [file 41467_2021_22630_MOESM4_ESM.zip › SourceData/README.html]

README.utf8


# Source data of the figures of ‘Linking functional traits to demographic parameters in high-diversity community models’

The folder contains the raw data of the figures, as well as the complete posterior distribution of the calibrated model.

posterior.csv - sample of the posterior distribution of the transfer function parameters. Each column is one of the parameters.

prior.csv - sample of the prior distribution of the transfer function parameters. Each column is one of the parameters.

Figure2A.csv - contains the predictive relative abundance (‘relAb’) of each species (“species”) for each unique temperature value (“temp”)

Figure2B.csv - contains the observed pseudo R2 values (“obs”) associated to each plot (“plot”). Are also indicated the associated temperature value (“temp”) and the 95% quantile of the distribution of the pseudo R2 values of the null models.

Figure3A.csv - contains the correlation value between the calibrated demographic rate parameters and each empirical functional traits. The column names indicate which pairs of demographic rates - functional traits the correlation value refers to. Each row represents a sample of the posterior distribution.

Figure3B.csv - contains the correlation value among the calibrated demographic rate parameters. The column names indicate which pairs of demographic rates the correlation value refers to. Each row represents a sample of the posterior distribution.

Figure4.csv - contains the demographic rate parameters values (‘Tmin’, ‘l’, ‘c’) for each species (‘species’) and the associated functional group (‘FG’)

FigureS1S2.csv contains the score of each species along the four first axes of the principal component analysis on species functional traits (columns Axis1, Axis2, Axis3, Axis4). It further contains species name (column SpeciesName), their functional group (column FG) and the average temperature across which the species was observed (TempNiche).

FigureS3.csv contains the source data for Figure S3. The column phi1 and phi2 contains the simulated values of the parameters \(\phi\_1\) and \(\phi\_2\) respectively. corT1y contains the correlation coefficient between the trait 1 and the y variable. corT2yT1 contains the partial correlation coefficient between the trait 2 and the y variable after factoring out the correlation with the trait 1. corT3yT1 contains the partial correlation coefficient between the trait 3 and the y variable after factoring out the correlation with the trait 1

FigureS4.csv contains the source data for Figure S4. corT1\_y\_prior1 contains the prior distribution of the correlation between trait 1 and y when \(\phi\_1\) is drawn in a uniform law (panel **a**); corT2\_y\_prior1 contains the prior distribution of the correlation between trait 2 and y when \(\phi\_1\) is drawn in a uniform law (panel **b**); corT3\_y\_prior1 contains the prior distribution of the correlation between trait 3 and y when \(\phi\_1\) is drawn in a uniform law (panel **c**); corT1\_y\_prior2 contains the prior distribution of the correlation between trait 1 and y when \(cos(\phi\_1)\) is drawn in a uniform law (panel **d**); corT2\_y\_prior2 contains the prior distribution of the correlation between trait 2 and y when \(cos(\phi\_1)\) is drawn in a uniform law (panel **e**); corT3\_y\_prior2 contains the prior distribution of the correlation between trait 3 and y when \(cos(\phi\_1)\) is drawn in a uniform law (panel **f**)

Figure S5 does not have a dedicated source data file but the source data is contained in posterior.csv and prior.csv.

FigureS6.csv contains the ROC curves for the different approaches. FPR contains the false positive rate values, TPR contains the true positive rate values and Model contains the name of the different models.

TabS1.csv contains the score of each functional trait along the four first axes of the principal component analysis on species functional traits. The last row of the table contains the total variance explained by each axes.

TabS4.csv contains the source data for the summary table Table S4.

TabS2S3S5.xlsx contains the source data for the table S2, S3, S5. Columns and rows are exactly the same as in the supplementary information.

TabS6.csv contains the summarized results of the fourth corner analysis. It contains the pearson correlation coefficient between each trait and the temperature gradient. The trait is stored in the column ‘Trait’, the coefficient value in ‘Obs’, the associated p-value of the permutation test in ‘Pvalue’ and the p-value adjusted for multiple tests in ‘Pvalue.adj’
